# Genome-Wide Characterization of Superoxide Dismutase (SOD) Genes in *Daucus carota*: Novel Insights Into Structure, Expression, and Binding Interaction With Hydrogen Peroxide (H_2_O_2_) Under Abiotic Stress Condition

**DOI:** 10.3389/fpls.2022.870241

**Published:** 2022-06-08

**Authors:** Roshan Zameer, Kinza Fatima, Farrukh Azeem, Hussah I. M. ALgwaiz, Muhammad Sadaqat, Asima Rasheed, Riffat Batool, Adnan Noor Shah, Madiha Zaynab, Anis Ali Shah, Kotb A. Attia, Muneera D. F. AlKahtani, Sajid Fiaz

**Affiliations:** ^1^Department of Bioinformatics and Biotechnology, Government College University, Faisalabad, Pakistan; ^2^Department of Biology, College of Science, Princess Nourah Bint Abdulrahman University, Riyadh, Saudi Arabia; ^3^Department of Botany, GC Women University, Faisalabad, Pakistan; ^4^Department of Agricultural Engineering, Khwaja Fareed University of Engineering and Information Technology, Rahim Yar Khan, Pakistan; ^5^Shenzhen Key Laboratory of Marine Bioresource and Eco-Environmental Sciences, College of Life Sciences and Oceanography, Shenzhen University, Shenzhen, China; ^6^Department of Botany, Division of Science and Technology, University of Education, Lahore, Pakistan; ^7^Department of Biochemistry, College of Science, King Saud University, Riyadh, Saudi Arabia; ^8^Department of Plant Breeding and Genetics, The University of Haripur, Haripur, Pakistan

**Keywords:** carrot, superoxide dismutase, reactive oxygen species, abiotic stress, expression pattern, phylogeny, comparative modeling, molecular docking

## Abstract

Superoxide dismutase (SOD) proteins are important antioxidant enzymes that help plants to grow, develop, and respond to a variety of abiotic stressors. SOD gene family has been identified in a number of plant species but not yet in *Daucus carota*. A total of 9 DcSOD genes, comprising 2 FeSODs, 2 MnSODs, and 5 Cu/ZnSODs, are identified in the complete genome of *D. carota*, which are dispersed in five out of nine chromosomes. Based on phylogenetic analysis, SOD proteins from *D. carota* were categorized into two main classes (Cu/ZnSODs and MnFeSODs). It was predicted that members of the same subgroups have the same subcellular location. The phylogenetic analysis was further validated by sequence motifs, exon–intron structure, and 3D protein structures, with each subgroup having a similar gene and protein structure. *Cis*-regulatory elements responsive to abiotic stresses were identified in the promoter region, which may contribute to their differential expression. Based on RNA-seq data, tissue-specific expression revealed that *DcCSD2* had higher expression in both xylem and phloem. Moreover, *DcCSD2* was differentially expressed in dark stress. All SOD genes were subjected to qPCR analysis after cold, heat, salt, or drought stress imposition. SODs are antioxidants and play a critical role in removing reactive oxygen species (ROS), including hydrogen peroxide (H_2_O_2_). DcSODs were docked with H_2_O_2_ to evaluate their binding. The findings of this study will serve as a basis for further functional insights into the DcSOD gene family.

## Introduction

Superoxide dismutases (SODs) are produced in almost all aerobic organisms, obligate anaerobes, and aero-tolerant anaerobes as a result of biological oxidations. These enzymes are found in almost all parts of the cell (Fink and Scandalios, 2002; Talukdar and Talukdar, 2013). Plant SOD proteins are a type of antioxidant enzyme that has been linked to protecting plants from the harmful effects of ROS and thus have a significant impact on plant growth, development, and their responses to abiotic stress (Rasul et al., 2017; Wang et al., 2017; Zhang et al., 2021a). In the natural environment, plants are frequently subjected to different environmental stressors, which result in the overproduction of ROS, such as singlet oxygen, hydrogen peroxide (H_2_O_2_), peroxyl radicals, hydroxyl radicals, and superoxide anion radicals. The controlled synthesis of cellular H_2_O_2_ serves a vital physiological function, but excessive amounts can have carcinogenic effects and induce cell death, and are produced in response to environmental stimuli in order to protect plant cells from O_2_ toxicity. Lowering its concentration can increase plant growth and quality, as well as antioxidant enzyme activity. This ROS causes degradation of cell membrane, macromolecules, and peroxidation, eventually leading to cell death and hence limiting crop production worldwide (Dehury et al., 2013). Plants have created a range of enzymatic as well as non-enzymatic defense systems to adapt to these ROS, including SOD system, glutathione reductase system, glutathione S-transferase system, ascorbate peroxidase (APX) system, glutathione peroxidase (GPX) system, peroxiredoxin (PrxR) system, dehydroascorbate reductase system, and catalase (CAT) system (Wang et al., 2017; Zhou et al., 2017; Lu et al., 2020). Metalloenzymes, commonly referred to as SOD, are the first line of defense for organisms against hazardous ROS. SODs catalyze the disproportionate conversion of O_2_ into oxygen and H_2_O_2_. Consequently, almost all green living cells are protected from the oxidative stress caused by aerobic circumstances (Dehury et al., 2013; Geng et al., 2018).

The SODs are divided into three classes based on their metal cofactors: copper/zinc SOD (Cu/Zn SOD or CSD), manganese SOD (MnSODs or MSD), and iron SOD (FeSODs or FSD) (Dreyer and Schippers, 2018). SOD proteins, which are encoded by nuclear genes, are found in several regions of the cell, with CSDs being found in the cytosol, chloroplast, and mitochondria (Feng et al., 2015; Verma et al., 2019). MSDs are thought to be found in peroxisomes as well as mitochondria, while FSDs are believed to be found in peroxisomes, chloroplast, and mitochondria (Verma et al., 2019). MSD and FSD have a high degree of sequence similarity, and most fungi and animals contain these two SODs, whereas there is no significant similarity in the case of CSD. Due to zinc and copper-binding as well as oxidation of intermolecular disulfide, CSD is generally highly stable. Copper is involved in superoxide disproportionation as a catalyst, while zinc and disulfide are involved in protein folding. Bacteria and plants exhibit all three types of SODs (Kliebenstein et al., 1998; Talukdar and Talukdar, 2013). In addition, *Streptomyces* were the first source for the identification, characterization, and cloning of a new form of SOD called nickel SOD or NiSOD. In contrast, plants have not been shown to contain these NiSODs (Hu et al., 2019).

The superoxide dismutase gene family has been found in a variety of plants, including *Arabidopsis* (Kliebenstein et al., 1998), *Zostera marina* (Zang et al., 2020), *Oryza sativa* (Dehury et al., 2013), *Gossypium hirustum* (Wang et al., 2017), grape (*Vitis vinifera L*.) (Guo et al., 2020), *Medicago truncatula* (Song et al., 2018), watermelon and melon (Zhang et al., 2021a), maize (*Zea mays* L.) (Sytykiewicz, 2014), and Rosaceae species (Li et al., 2021). Studies on different plant SOD genes have shown that these can act as the first line of defense against abiotic stimuli including heat, cold, drought, and salinity (Zhang et al., 2021a,b). The expression pattern of individual SOD gene family members is different in different species. This expression of individual members also varies significantly according to environmental stresses. In *A. thaliana*, the level of transcription of chloroplastic *FSD1* and *CSD2* were reduced by ozone fumigation, while chloroplastic *FSD2* mRNAs remained relatively constant. However, *FSD2* mRNA increased considerably in response to UV-B, but *FSD1* and *CSD2* remained stable. Furthermore, *CSD1* may be involved in response to both UV-B and ozone illumination (Kliebenstein et al., 1998). In legumes, the overexpression of antioxidant enzymes may provide additional expression throughout the N_2_ fixation, particularly under stress and senescence. Overexpression of *MnSOD* from *Nicotiana plumbaginifolia* protected alfalfa plants from water deprivation. Furthermore, overexpression of *MnSOD* in plants improved *FeSOD* activity (Kuzniak, 2002; Rubio et al., 2002).

Under drought stress, transgenic potato (carrying *CSD* cloned from *Potentilla atrosanguinea*) demonstrated higher stomatal conductance and photosynthetic rates as compared to the other wild-type plants. Similarly, more transcripts were produced by the white clover *FSD, MSD*, and *CSD* genes in drought stress. While banana SOD genes were downregulated, SOD gene expression from tomatoes was altered (Pal et al., 2013; Feng et al., 2015, 2016; Zhang et al., 2015). The expression of three types of SOD genes showed significant upregulation in *G*. *hirsutum* during drought stress; however, only the expression of CSD genes was increased under cold stress and the soluble sugar and photosynthetic rate were upregulated. Under cold stress, the concentration of ROS was higher with decreased SOD activity in tea plants, and damage to leaves was more severe in cold-sensitive cultivars than in cold-resistant cultivars (Zhang et al., 2014; Zhou et al., 2019). In wheat, members of this family showed different expressions in various tissues. Under heat, drought, and salt stress, wheat CSDs and MSDs were highly upregulated (Zafra et al., 2018). In *Z. marina*, all the five SOD genes were expressed in various tissues (leaf, root, male flower, female flower—early and late tissues). Consequently, boosting SOD activity is one of the most effective strategies for plants to withstand a variety of abiotic stresses (Talukdar and Talukdar, 2013).

*Daucus carota* (carrot) subsp. *Carota* L (2n = 2x = 18) is one of the most important economical crops globally and the most important member of the Apiaceae family. *D. carota* is one of the most essential vegetables in the world, and it comes in a variety of colors, including yellow, red, orange, white, and purple-pink, with its root rich in alpha and beta-carotene and a good source of vitamin K and B6 (Zhou et al., 2019). The most well-known feature of *D. carota* is that it was the first species to successfully demonstrate the integrity of living cells. Furthermore, experiments provided clues that altering DNA levels per cell leads to differential genome organization. Similarly, the significance of plastid transformation has been proven in several metabolic, biochemical, electron-microscopic, and histochemical studies. This reproducible system was crucial in elucidating the connection between genome organization and growth regulators and is still used for recent molecular biology research. Similarly, cell programming in *D. carota* is particularly simple at the tissue and cellular levels. These features make *D. carota* an ideal system for fundamental studies.

Superoxide dismutase genes help enhance food production by resisting a variety of environmental challenges (salt, drought, alkali, light/dark, and cold stress). Furthermore, the evolution and diversity in this gene family may lead to functional diversity, which may help to further understand the carrot stress-responsive genes. Therefore, to address whether SOD genes are conserved in *D. carota*, we identified these genes in carrots. In this study, we determined the functional diversity and expression profiles of *DcSOD* genes, which provides useful information for stress-resistant carrot breeding. This comprehensive investigation of the *DcSOD* genes will also serve as a foundation for future research into the molecular functions of *DcSODs*.

## Materials and Methods

### Database Search and Sequence Retrieval

The full-length SOD protein sequences for *A. thaliana* were obtained and downloaded from the NCBI (https://www.ncbi.nlm.nih.gov/) (Jenuth, 2000). The SOD gene family members in *D. carota* were identified using these sequences as queries in the BLASTP search. All members of the *D. carota* SOD family had their proteins sequences retrieved from NCBI. Pfam (https://pfam.xfam.org/) (Bateman et al., 2004), HMMER (http://hmmer.org/) (Finn et al., 2011), CDD (https://www.ncbi.nlm.nih.gov/Structure/cdd/wrpsb.cgi) (Marchler-Bauer et al., 2011), and InterPro (https://www.ebi.ac.uk/interpro/) (Hunter et al., 2009) were used to check for specific domains in putative sequences, removing any that did not contain conserved domains which are required for SOD proteins to function properly. After editing manually the remaining protein sequences to remove redundancy, they were considered candidate SOD proteins. Domain architecture was constructed using TBtools (Chen et al., 2020). NCBI database was used to obtain chromosomal localization, CDS length, and exon number (Brown et al., 2015). The online ProtParam ExPASY tool (https://web.expasy.org/protparam/) was used to calculate the physical as well as chemical characteristics of *DcSOD* proteins, including the number of protein length (aa), molecular weight (Da), theoretical isoelectric point (pI), and grand average of hydropathicity (GRAVY) (Gasteiger et al., 2005). In addition, by using the online WoLF PSORT (https://wolfpsort.hgc.jp/) program, the subcellular localization of *DcSOD* genes was predicted (Horton et al., 2007).

### Multiple Sequence Alignment, Phylogenetic Analysis, Conserved Motifs, and Gene Structure Analysis

To determine the evolutionary relationship of SOD proteins from *D. carota* and diverse plant species, a total of 42 protein sequences of SODs from *D. carota, A. thaliana* (Kliebenstein et al., 1998)*, C. sinensis* (Zhou et al., 2019), *O. sativa* (Dehury et al., 2013), and *S. miltiorrhiza* (Han et al., 2020) were used. The ClustalW program was used for multiple sequence alignment of these SOD proteins with default parameters, and a maximum-likelihood (ML) tree was constructed by using MEGA7 software (https://www.megasoftware.net/) (Kumar et al., 2016), with a bootstrap value of 1,000, and was edited online using iTOL: Interactive Tree Of Life (https://itol.embl.de/) (Letunic and Bork, 2021). The Multiple EM for Motif Elicitation (MEME) tool (https://meme-suite.org/meme/) (Bailey et al., 2015) was used to determine the conserved motifs in each SOD protein sequence. The maximum motif number was set to 10, while the rest of the parameters were left at their default values. The Gene Structure Display Server (GSDS) (http://gsds.gao-lab.org/) (Hu et al., 2015) was used to predict gene intro/exon structure using coding and genomic sequences of *D. carota, O. sativa*, and *A. thaliana* as input data.

### Chromosomal Distribution, Gene Duplication Events, and Syntenic Analysis

The location of each SOD gene in *D. carota* was identified using the NCBI gene database (https://www.ncbi.nlm.nih.gov/gene/) (Brown et al., 2015). All SOD members were physically mapped on *D. carota'*s nine chromosomes. The genes that possessed ≥80% sequence identity were considered duplicated genes. The value of synonymous substitution (Ks), as well as non-synonymous substitution (Ka) of duplicated gene pairs, was calculated using the DnaSP version 6 offline tool (Rozas et al., 2017). The ratio (Ka/Ks) was used in the calculation of the selection pressure that aided the gene family's evolution. The following formula was used for calculating the duplication time of duplicated genes: T = Ks/2λ × 10^−6^ Mya (where λ represents substitution per synonymous site per year and is equal to 1.5 × 10^−8^ for dicots) (Lynch and Conery, 2000). A genetic relationship map of chromosomes and duplicated gene pairs was visualized by using TBtools Advance Circos (Chen et al., 2020).

### 3D Structure Prediction, Molecular Docking Analysis, and PPI Analysis of *DcSODs*

To precisely understand the functions of a protein, its 3D structure is important. Thus, the 3D structure of *DcSODs* was predicted by the I-TASSER server (https://zhanggroup.org/I-TASSER/) (Zhang, 2008). Moreover, the SAVES server (https://servicesn.mbi.ucla.edu/SAVES/) was used to verify the models (Elshemey et al., 2010). The 3D structures were visualized using the UCSF Chimera visualization tool (Pettersen et al., 2004). The 3D structure of ROS, i.e., H_2_O_2_, against PubChem (https://pubchem.ncbi.nlm.nih.gov/) IDs (784) were retrieved (Kim et al., 2016), and then molecular docking analysis was done using PatchDock server (https://bioinfo3d.cs.tau.ac.il/PatchDock/) (Schneidman-Duhovny et al., 2005). After the successful docking of the H_2_O_2_ ligand against all nine *DcSODs*, interaction analysis of protein–ligand complexes was carried out one by one using the UCSF Chimera window version 1.6 (Pettersen et al., 2004). The possible protein–protein interactions of these DcSOD proteins were predicted using the STRING database (http://string-db.org/) (Szklarczyk et al., 2019).

### *Cis*-Regulatory Elements and Expression Pattern of Transcriptome Analysis

To analyze the potential functions of *DcSODs*, a 1,000 bp upstream sequence of the initiation codon of each *DcSOD* gene was retrieved from the NCBI nucleotide database (https://www.ncbi.nlm.nih.gov/nucleotide/) (Orlek et al., 2017). To examine the *cis*-elements in these promoter sequences, the PlantCARE server (http://bioinformatics.psb.ugent.be/webtools/plantcare/html/) (Rombauts et al., 1999) was used, and the results were visualized using TBtools (Chen et al., 2018). To gain insight into the tissue-specific expression patterns of *DcSOD* genes, RNA-Seq data (BioProject: PRJNA610539) and under light/dark stress (BioProject: PRJNA626692) was obtained from SRA-NCBI (https://www.ncbi.nlm.nih.gov/sra) (Sherry et al., 2012). A heat map was generated based on the reads per kilobase per million (RPKM) values of individual genes in taproot tissue samples. DcSOD gene expression levels analyzed in taproot of *D. carota* genotypes were Purple 68 and Purple Haze. The heat maps were illustrated using TBtool (Chen et al., 2020).

### Plant Growth and Treatments

The plants were grown in a growth chamber for 2 weeks under controlled conditions: 25 ± 2°C temperature and 65% relative humidity. Then, the plants were shifted to different incubators for stress imposition. In the 1^st^ incubator, the temperature was set at 4°C for cold stress treatment. In the 2^nd^ incubator, the temperature was increased up to 42°C for heat stress treatment. In the 3^rd^ incubator, plants were treated with 200 mmol/L NaCl for salt stress treatment. For drought stress treatment, 2-week-old plants were grown under water-limiting conditions. These plants were harvested in two groups for RNA extraction. In group A, plants were harvested at 0, 06, 12, and 24 h for cold, heat, and salt stress. While in group B, plants were harvested at 0, 1, 3, and 6 days for drought stress. These samples were immediately frozen in liquid nitrogen and kept at −80°C until needed.

### Validation of Quantitative Real-Time PCR (qRT-PCR)

TRIzol reagent was used to extract RNA from leaf samples as directed by the manufacturer and quantified using a NanoDrop spectrophotometer. Using a cDNA synthesis kit, 1 μg of total RNA was reverse transcribed with dsDNase (Maxima H Minus First-Strand). 10 × dsDNase buffer, total RNA, dsDNase, and nuclease-free H_2_O were added to an RNase-free tube on ice to obtain a total volume of 10 μl of total RNA. The mixture was gently mixed and spun before being incubated at 37°C for 2 min in a hot water bath and then stored on ice. The synthesis chemicals of the first strand (5 × RT buffer, 10 mM dNTP mix, oligoT primer, and maxima H minus reverse transcriptase enzyme) were then added and gently mixed before being centrifuged briefly. The mixture was then incubated for 30 min at 50°C and 5 min at 85°C. Finally, the mixture was kept at a temperature of around −80°C until it was used. iTaq Universal SYBR Green SuperMix and Real-time PCR detection system (CFX96 Touch™ Real-Time PCR Detection System) were used to carry out qRT-PCR. Actin-7 was considered a housekeeping gene and used as an internal control. Primers were designed using the online NCBI Primer-BLAST Program (https://www.ncbi.nlm.nih.gov/tools/primer-blast/), and their specificity was verified using an online tool Oligo Calculator (http://mcb.berkeley.edu/labs/krantz/tools/oligocalc.html). Student's *t*-test (*p* < 0.05 = > ^*^, *p* < 0.01 = >^**^) was performed to compare treatment with control and calculate the significance level.

## Results

### Characterization of *DcSODs* in *D. carota*

**Figure 1:**
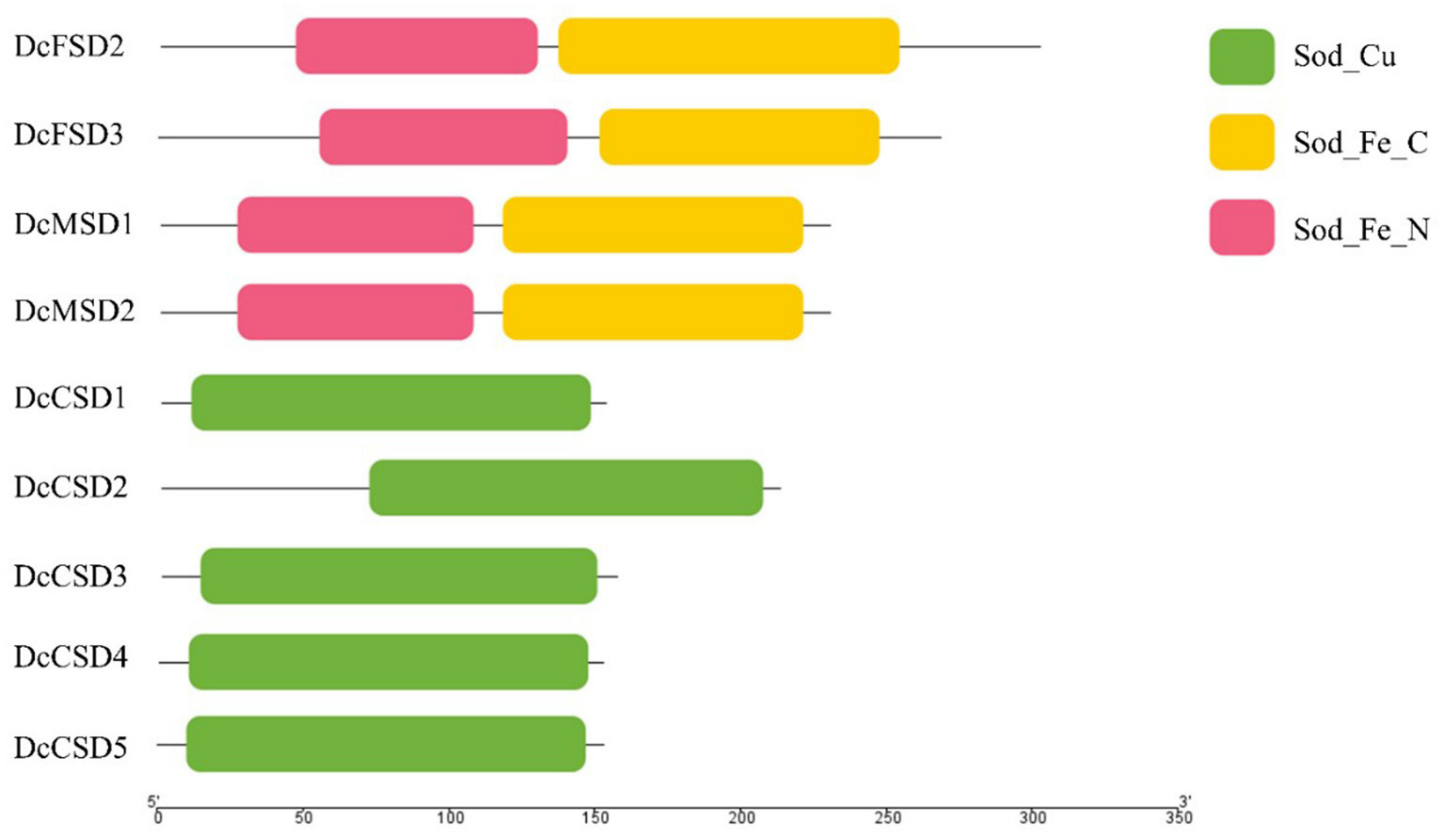
Symbolic domain structure of *Daucus carota* superoxide dismutases.

A total of 7 *A. thaliana* SOD genes were used as queries in the BLASTp search to get a complete overview of the *D. carota* SOD gene family. The accessions missing the SOD-specific domains were excluded from the studies. Finally, in the *D. carota* genome, 9 SOD proteins comprising specific domains were identified (Figure 1, Supplementary Table S1). These 9 SOD proteins included two DcFSDs (*DcFSD2*−*3*), two DcMSDs (*DcMSD1-2*), and five Cu/*ZnSODs* (*DcCSD1-5*). The C-terminal iron/manganese superoxide dismutase domain and alpha hairpin domain were found in both putative DcFSDs and DcMSDs. The copper/zinc superoxide dismutase domain was present in all DcCSDs, which is a characteristic feature of Cu/*ZnSODs*.

By computing different parameters, the biochemical and physiological properties of the nine DcSODs were determined (Table 1). The length of these proteins was found between 152 to 301 amino acids (aa), with an average length of 205 aa. Accordingly, the molecular weight ranged from 15.01 to 34.53 kDa. According to earlier research, all Cu/ZnSODs are acidic, whereas Fe-MnSODs are either acidic or basic (Zhou et al., 2017). In this study, the majority of the DcSODs were acidic with pI values ranging from 5.1 to 7.83. Furthermore, the DcSOD proteins' predicted GRAVY values were negative, indicating that they are hydrophilic. The prediction regarding subcellular localization showed that most of the DcCSDs were localized in the cytoplasm, except for the *DcCSD2*, which was localized in the chloroplast. In addition, DcFSDs and DcMSDs were localized in chloroplast and mitochondria, respectively.

**Table 1:** Features of SOD genes in *Daucus carota*.

**Gene name**	**Gene symbol**	**Chr**	**Start**	**End**	**CDS**	**Exon**	**Protein length (aa)**	**Molecular weight (Da)**	**Isoelectric point (PI)**	**Grand average of hydropathy (GRAVY)**	**Cell location**
*DcFSD2*	LOC108220279	5	26,243,171	26,246,462	1,351	9	301	34,528.75	5.1	−0.618	Chloroplast
*DcFSD3*	LOC108207726	1	31,055,434	31,060,777	1,182	9	268	30,972.39	7.09	−0.387	Chloroplast
*DcMSD1*	LOC108223791	5	41,938,799	41,944,971	1,046	6	229	25,427.87	6.71	−0.304	Mitochondrial
*DcMSD2*	LOC108215224	3	23,817,274	23,825,843	1,056	6	229	25,260.72	7.83	−0.273	Mitochondrial
*DcCSD1*	LOC108200536	9	27,501,162	27,504,082	846	8	152	15,011.48	5.46	−0.203	Cytoplasmic
*DcCSD2*	LOC108219531	4	11,280,322	11,285,715	886	8	212	21,635.52	5.78	0.03	Chloroplast
*DcCSD3*	LOC108222825	5	10,957,249	10,965,078	777	7	156	15,862.58	6.09	−0.178	Cytoplasmic
*DcCSD4*	LOC108226548	1	29,300,467	29,303,783	865	8	152	15,025.51	5.46	−0.202	Cytoplasmic
*DcCSD5*	LOC108212562	1	37,839,678	37,841,990	712	8	153	15,841.56	5.95	−0.422	Cytoplasmic

### Phylogeny, Conserved Motifs, and Gene Structure

**Figure 2:**
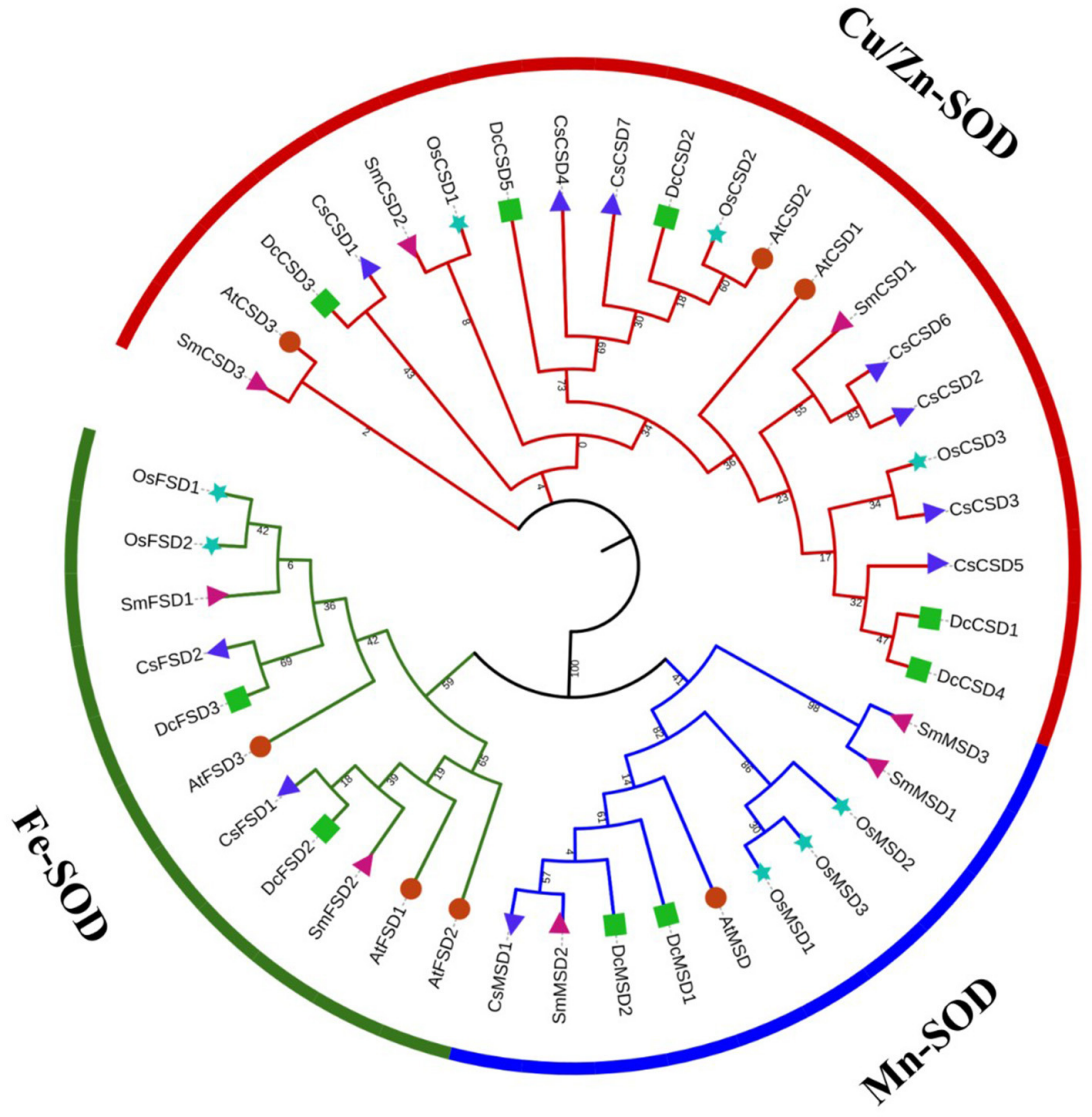
Phylogenetic tree analysis of SOD family genes of *D. carota* and other species. In this tree, different symbols are representing different plants: green square for *D. carota*, powder-blue star for *Oryza sativa*, red circle for *Arabidopsis thaliana*, blue triangle for *Camellia sinensis*, and pink triangle for *Salvia miltiorrhiza*. Three different strips represent the different groups. The phylogenetic tree was constructed using the MEGA7 maximum likelihood method.

To gain insight into the evolutionary relationship of DcSOD proteins, an ML phylogenetic tree was constructed using 42 full-length proteins sequences (nine DcSODs, seven AtSODs, eight OsSODs, eight SmSODs, and ten CsSODs). The DcSOD proteins were clustered into three distinctive subfamilies, namely, CSDs, MSDs, and FSDs, which were found to be in good accordance with their metal cofactor types. The Cu/Zn-SODs (colored red) constituted the largest group with 21 members. In another larger clade, Fe-SODs were clustered with Mn-SODs (Figure 2), indicating that these two groups originated from the same ancestor (Wang et al., 2017).

**Figure 3:**
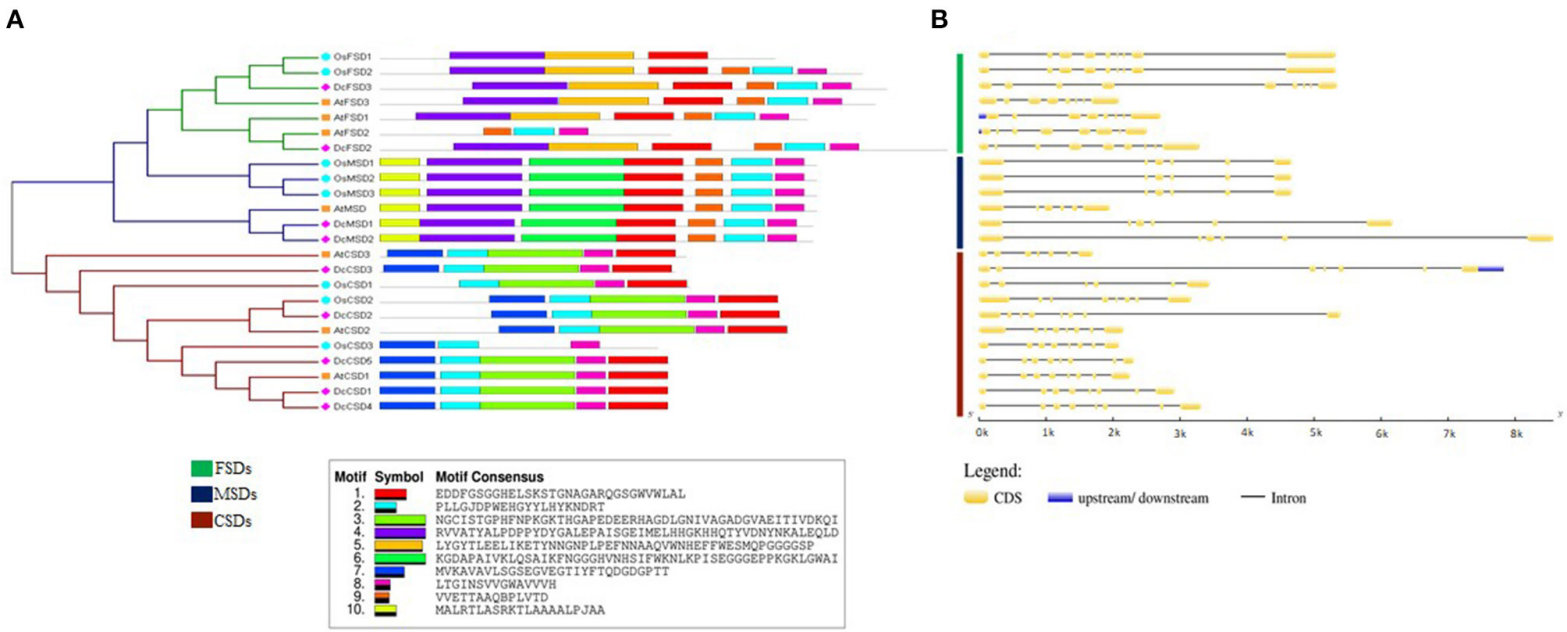
**(A)** Conserved motif and **(B)** gene structure analysis of SOD family genes in *D. carota*.

To reveal similarities of *D. carota* SOD proteins in each subfamily, the gene structure of DcSODs and their conserved motifs were analyzed. Members of the same subfamily shared common conserved motifs. Motif 7 was conserved among all the SOD proteins. Similarly, motif 1 was also conserved in almost all proteins. All members of subfamily FSD had motifs 7, 5, 1, 2, 9, and 8, except for *AtFSD2* having only 3 motifs (motifs 1, 7, and 5 were absent). All proteins of subfamily MSD had motifs 1, 6, 7, 10, 2, 8, and 9. Motifs 7, 2, 3, 8, and 1 were present in all members of the CSD group, except for *OsCSD1* and *OsCSD2* in which motifs 7 and 3 were missing, respectively (Figure 3A). All of the protein subfamilies had nearly identical motif compositions, implying that there was no major divergence in function or sequence. Similarly, in general, the members which belonged to the same subfamily had nearly comparable intron and exon organization patterns (Figure 3B). For example, each gene in the FSD subfamily had at least eight conserved exons. In contrast, MSD subfamily members had a relatively small number of exons, with six exons in each gene. The gene structure of subfamily CSD members was quite different, with the exon numbers ranging from six to eight, but the six exons were conserved in each gene. However, the gene structures of the similar subgroup were generally conserved.

### Chromosomal Mapping and Gene Duplication

**Figure 4:**
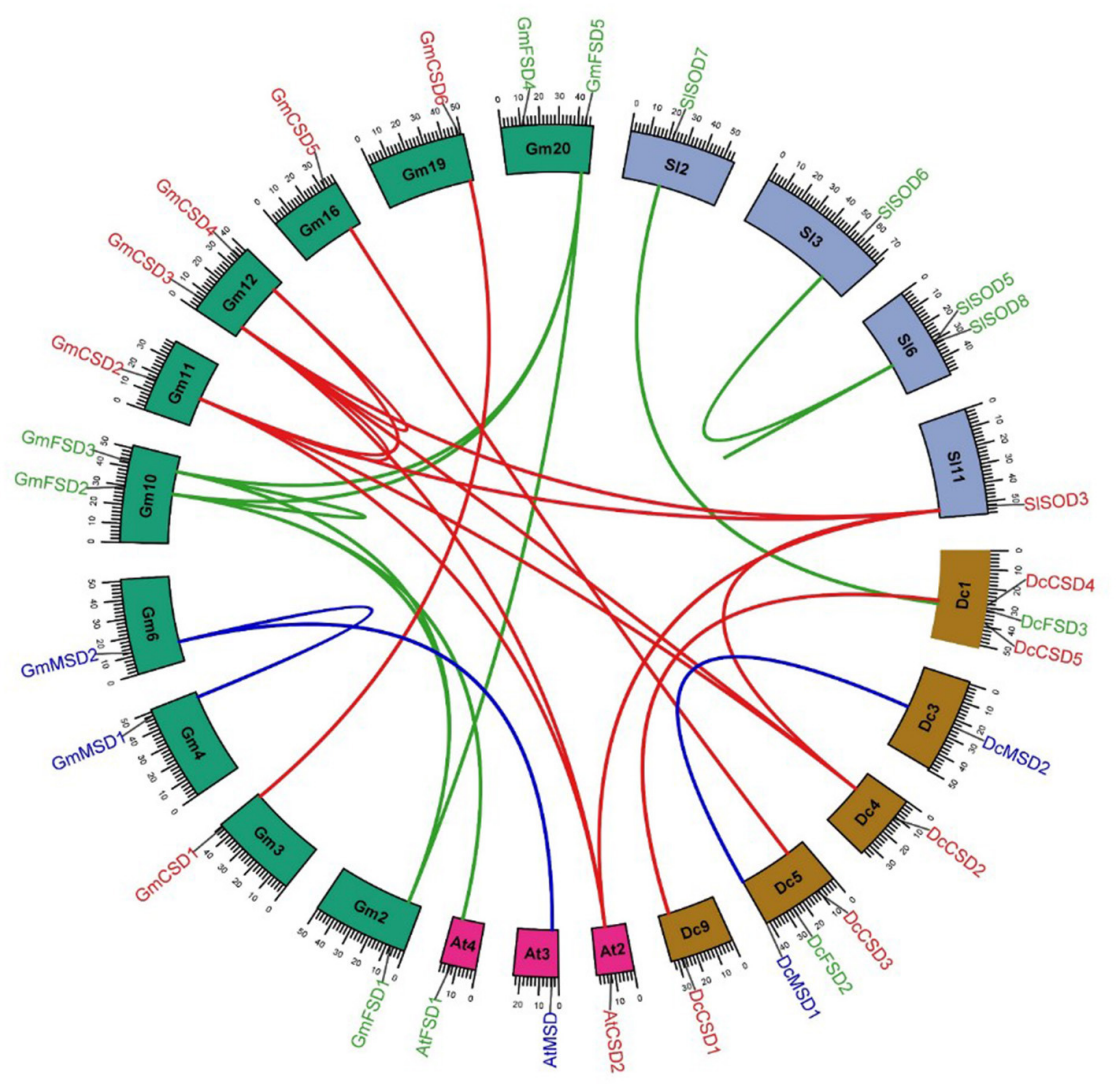
Syntenic analysis of SOD family genes in *A. thaliana, D. carota, G. max, S. lycopersicum*, and *S. bicolor*. Blocks are representing the chromosomes and the duplicated genes are connected by lines.

To analyze the genomic distribution of *DcSODs* genes, the chromosomal gene location and duplication events were identified using syntenic analysis. The *D. carota* genes were randomly distributed on 5 out of 9 chromosomes. Dc1 and Dc5 chromosomes had 3 genes each, while Dc3 and Dc4 had only one gene on each. Furthermore, no SOD genes were found on Dc2, Dc6, Dc7, and Dc8 (Figure 4). According to duplication analysis, *DcMSD*1/*DcMSD*2 and *DcCSD*1/*DcCSD4* are the two duplication pairs that resulted from segmental duplication. To analyze the relationships between the SOD genes and gene duplications, we identified the syntenic blocks of SOD genes among *D. carota, A. thaliana, G. max*, and *Solanum lycopersicum* (Figure 4). Segmental duplication events may promote better regulation of SOD activities through functional divergences under stress conditions, as well as spatially specific and temporal expression of these genes [61]. Furthermore, no tandem duplication was found in DcSODs. The syntenic analysis regarding cross-genome indicated that three GmSODs and two SlSODs genes had orthologous genes in *D. carota* genome (Figure 3, Supplementary Table S2). These findings indicate that segmental duplication is a possible factor in the expansion of DcSOD genes.

These duplicate non-synonymous rates (Ka), synonymous rates (Ks), and Ka/Ks were determined, and the time of duplication was estimated using Ks values. The Ks of these two segmental duplicates ranged from 0.33 to 0.64. Thus, the divergent time ranged from 7.81 Mya to 12.53 Mya. The Ka/Ks value of *DcMSD*1/*DcMSD*2 was less than 1, indicating that purifying selection occurred in this duplication. In contrast, *DcCSD*1/*DcCSD4* had a Ka/Ks value of more than 1, thus it experienced positive selection (Supplementary Table S2) (Roth and Liberles, 2006).

### Conserved Exon Analysis

**Figure 5:**
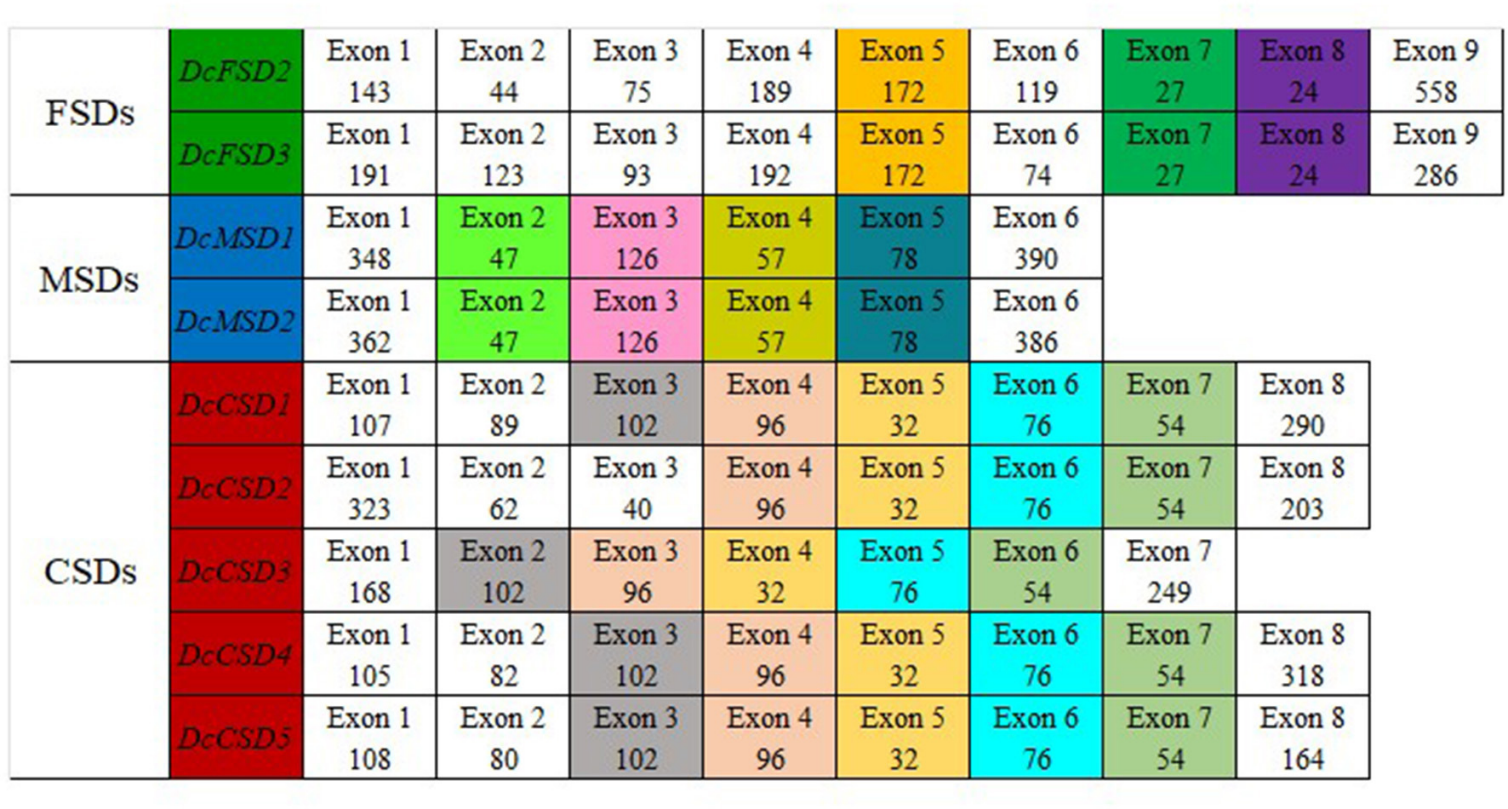
Splice site analysis of 9 members of *D. carota* SOD gene family. Exons colored the same in each group conserved in length in that particular group.

According to this analysis, each subfamily has its own set of conserved exons. The FSD group with two members *DcFSD2* and *DcFSD3* has three highly conserved exons (172, 27, and 24 nt). The MSD group with two members *DcMSD1* and *DcMSD2* has four preserved exons (47, 126, 57, and 78 nt). The CSD group contains five members (*DcCSD*1/ *DcCSD5*) and five preserved exons. *DcCSD*1, *DcCSD*3, *DcCSD*4, and *DcCSD*5 have four preserved exons (96, 32, 76, and 54 nt). The remaining exon (102) is also conserved among these genes, except *DcCSD*2, which does not have this exon (Figure 5). The fact that these exons have been preserved indicates that they are the products of duplication events.

### Protein 3D Structure Prediction

**Figure 6:**
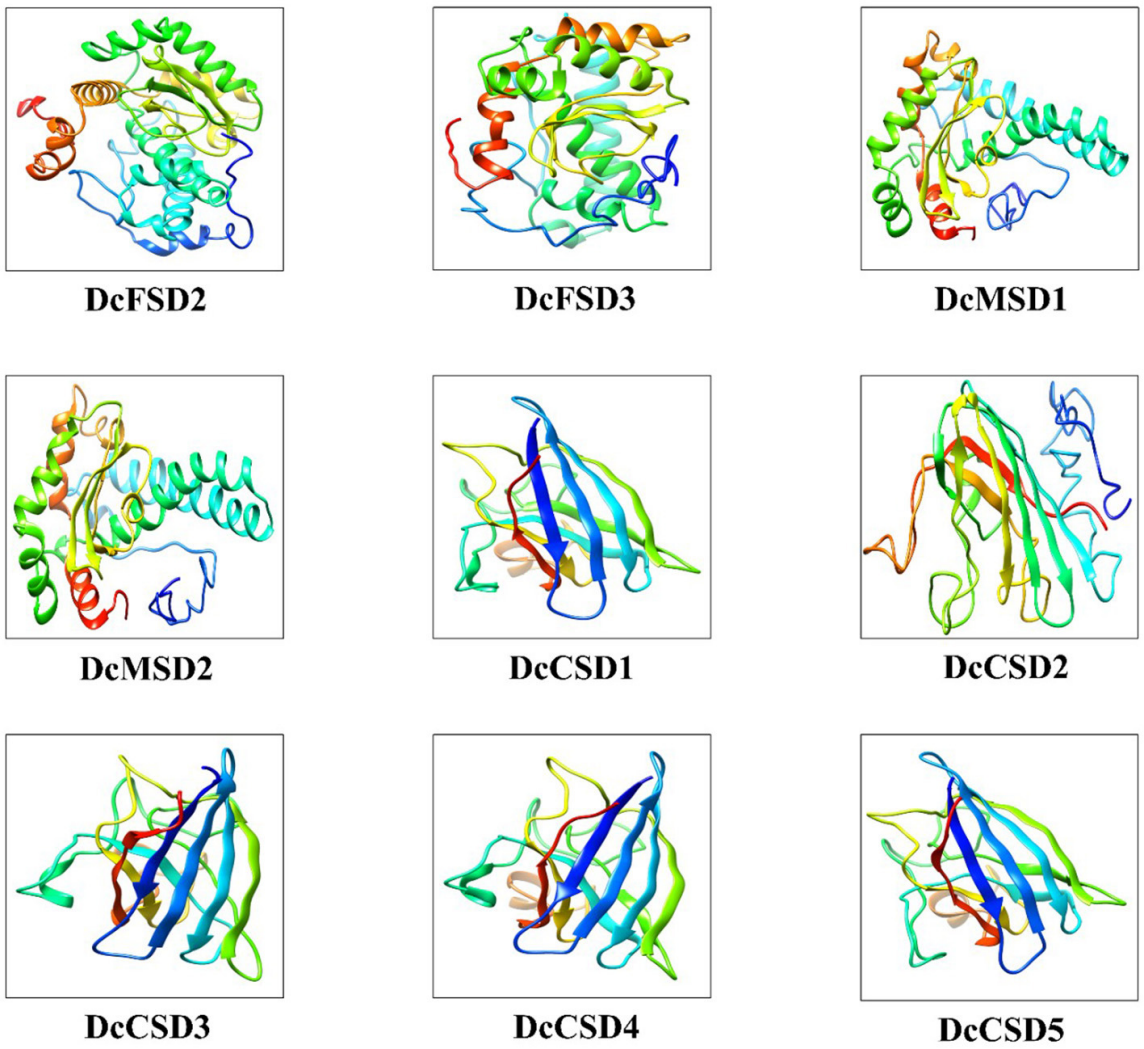
The 3D structure modeling of DcSOD proteins. These are the final models predicted with different colors indicating different helices, sheets, and domains.

Proteins are difficult to describe as they are complex chemical entities with a large number of convolutes topology and variable atoms. In this study, three-dimensional models of 9 DcSOD proteins were predicted by using the I-TASSER server. The resulting models had good stereochemical characteristics both globally and locally. The yellow color represents the helices, the green color represents the sheets or strands. Proteins belonging to particular groups have similar structural symmetry. Members of the MSD and the FSD subfamily have an almost similar structure with the same number of helices and sheets, while proteins from the subfamily CSD have an almost similar structure (Figure 6).

### Molecular Docking Analysis of DcSODs With H_2_O_2_

**Figure 7:**
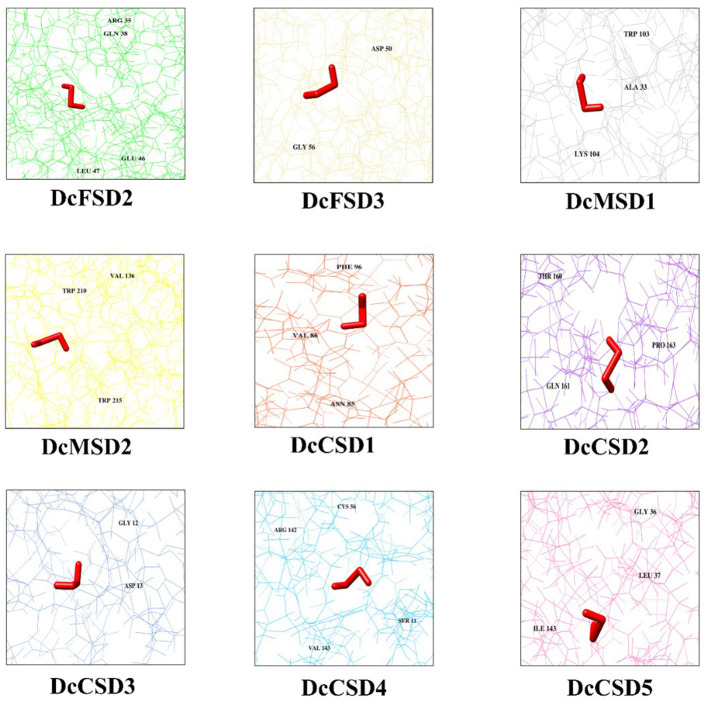
H_2_O_2_ docked against 9 DcSODs. The different color background of the diagram represents protein residues, conserved residues of the protein are also labeled, and the prominent red color represents the ligand (H_2_O_2_).

Molecular docking of H_2_O_2_ was performed against 9 DcSODs to check the binding as SODs are antioxidants and play a vital role to remove the ROS including H_2_O_2_ (Gill and Tuteja, 2010). Molecular docking results showed a good binding ability among all the DcSODs (Figure 7). Docking complexes have −3.69 kcal/mol (*DcCSD4*) to −1.94 kcal/mol (*DcFSD3*) global energy and −3.28 kcal/mol (*DcCSD2*) to c1.41 kcal/mol (*DcCSD3*) attractive Van Der Waals (VDW) energy that is a good score. Conserved residues of *DcFSD2* are ARG 35, GLN 38, GLU 46, and LEU 47, and their bond distances are 2.998, 2.594, 2.584, and 3.067 Å, respectively. Conserved residues of *DcFSD3* are ASP50 and GLY 56 with bond distances of 2.460 and 2.324 Å. *DcMSD1* has three conserved residues ALA 33, TRP 103, and LYS 104 with 2.843, 2.269, and 2.329 Å bond distances. Conserved residues of *DcMSD2* are TRP 210, TRP 215, and VAL 136, and their bond distances are 2.518, 2.159, and 2.772 Å, respectively. *DcCSD1* has three conserved residues ASN 85, VAL 86, and PHE 96 with bond distances of 2.792, 2.240, and 2.848 Å. Residues THR 160, GLN 161, and PRO 163 are the conserved residues of *DcCSD2* with bond distances of 3.085, 2.870, and 2.377 Å, respectively. *DcCSD3* has only conserved residues GLY 12 and ASP 13 with bond distances of 2.098 and 1.086 Å. *DcCSD4* has four conserved residues SER 11, CYS 56, ARG 142, and VAL 143, and their bond distances are 2.356, 3.536, 2.793, and 3.138 Å, respectively. *DcCSD5* has three conserved residues GLY 36, LEU 37, and ILE 143, and their bond distances are 3.108, 2.329, 2.363 Å, respectively (Supplementary Table S3). Based on the predicted structures and docking analysis of binding sites, protein–protein interaction (PPI) analysis was also performed to further explore the potential common function of interacted proteins (Supplementary Figure S1).

### *Cis*-Regulatory Element Analysis in Promoter Sequences of DcSODs

**Figure 8:**
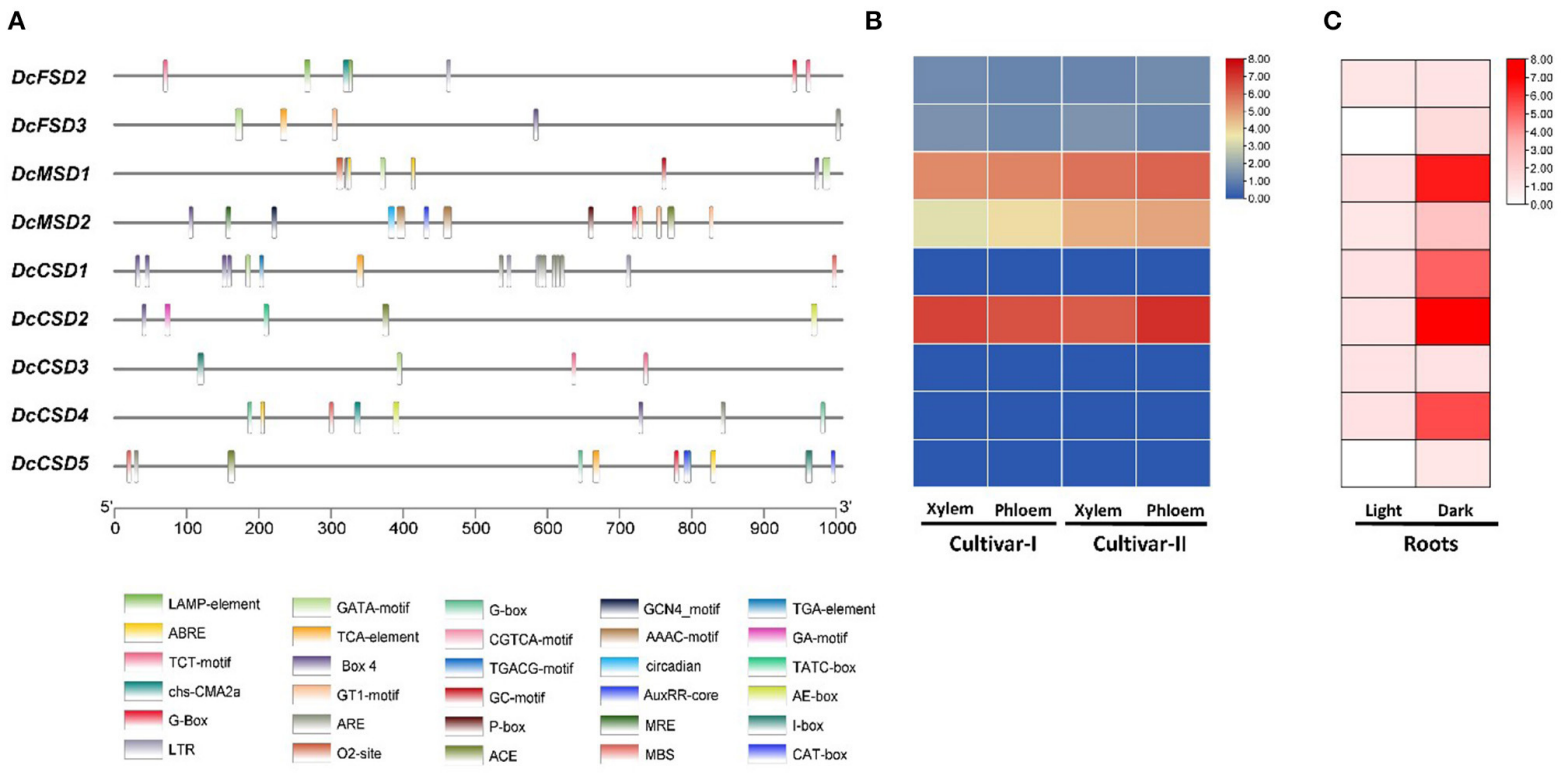
**(A)** *Cis*-regulatory elements in upstream 1 kb region of *D. carota* SOD coding sequences. Each element is given a specific color and depicts the presence of that element in genes. **(B)** Expression analysis of DcSOD gene family. Heatmap expression profiles of the genes in xylem and phloem tissues. **(C)** Expression analysis of DcSODs in roots under light/dark stress.

To better understand the role of *DcSOD* under diverse stresses, *cis*-regulatory elements in the promoter sequences of *DcSOD* genes were studied. According to the results, abiotic stress-related elements and hormone-responsive *cis*-regulatory elements were present abundantly in the promoter sequence of DcSOD gene family (Supplementary Table S4). Four major *cis*-element classes have been identified, namely, light-responsive, stress-responsive, hormone-responsive, and MYB-binding sites (Figure 8A). All the promoters had several light-responsive *cis*-elements, particularly *DcCSD4, DcCSD5*, and *DcMSD2* containing 13, 10, and 10 elements, respectively. *Cis*-regulatory elements having involvement in meJA-responsiveness also existed in the promoter regions of *DcCSD5* and *DcMSD1*, with 4 and 2 *cis*-elements, respectively. ABA-responsive elements, which are hormone-responsive elements, occurred in *DcFSD2, DcMSD1, DcMSD2, DcCSD4*, and *DcCSD5*. The promoters of *DcCSD2* and *DcMSD2* also contained the gibberellins-responsive elements. *DcCSD2* and *DcFSD1* also had elements that showed responses in low temperatures. The promoters of *DcCSD4, DcCSD5*, and *DcCSD1* possessed MYB binding sites, which were involved in drought inducibility. *DcMSD2* possesses MYB binding site elements that were involved in light responsiveness. Moreover, *cis*-elements necessary for anaerobic induction were also present in some gene promoter regions (Supplementary Table S4). These findings suggested that the SOD gene family was important in plant abiotic stress response as well as development.

### Expression Profiles of DcSODs

RNAseq analysis was performed in *D. carota* tissues, namely, Purple 68 (xylem and phloem) and Purple Haze (xylem and phloem), and the expression profiles of these DcSODs were visualized. The results show that five genes (*DcFSD2, DcFSD3, DcMSD1, DcMSD2*, and *DcCSD2*) are expressed in xylem and phloem *of D. Carota*, while the remaining four genes (*DcCSD1, DcCSD3, DcCSD4*, and *DcCSD5*) have no expression. Out of five, three genes (*DcCSD2, DcMSD1*, and *DcMSD2*) have higher expression, while the other two (*DcFSD2 and DcFSD3*) have a minor expression (Figure 8B). To determine the expression of these DcSODs in roots under abiotic stress (light/dark), RNA-seq analysis was performed. The results show that under dark stress, four genes (*DcMSD1, DcCSD1, DcCSD2*, and *DcCSD4*) were highly upregulated, while the other five genes (*DcFSD2, DcFSD3, DcMSD2, DcCSD3*, and *DcCSD5)* were slightly upregulated. Under light stress, seven genes (*DcFSD2, DcMSD1, DcMSD2, DcCSD1, DcCSD2, DcCSD3*, and *DcCSD4)* were slightly upregulated, while two genes (*DcFSD3 and DcCSD5*) had no expression (Figure 8C).

### Expression Validation of DcSOD Genes Through qRT-PCR

**Figure 9:**
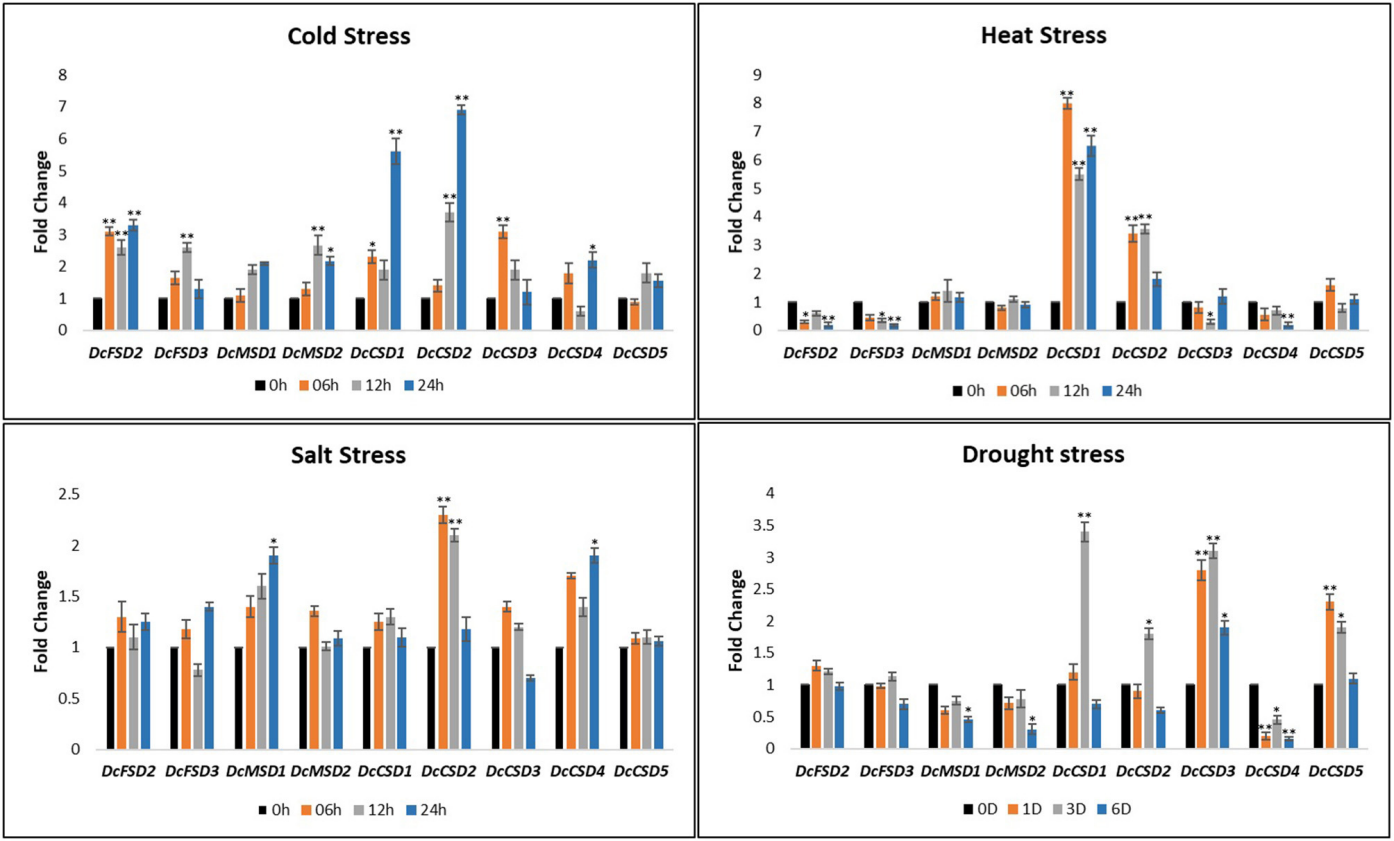
Relative qRT-PCR expression verification of *DcSODs* genes under cold, heat, salt, and drought stress. In nontreated plants, one (01) was the default expression value for each gene. Significant change in expression is indicated by an asterisk (*p* < 0.05 = > *, *p* < 0.01 = >**).

To predict the molecular role of DcSOD genes in *D. carota*, a qRT-PCR was performed on the leaves under various abiotic conditions including cold, heat, salt, and drought stress. The expression level of a few genes was increased under these stresses (*DcCSD1, DcCSD2, DcCSD5*, and *DcFSD2*). For cold stress, the expression level of most of the transcripts was increased after 6 h of treatment. After maximum treatment, some gene transcripts (*DcCSD1, DcCSD2*, and *DcFSD2*) were highly expressed. Almost all DcSOD genes had their expression increased after cold stress. In heat stress, a variation in expression level was observed. Some transcripts including *DcFSD2, DcFSD3*, and *DcCSD4* had their expression decreased. *DcCSD1* and *DcCSD2* were highly upregulated under heat stress. Salt stress also resulted in higher expression of most of the transcripts including *DcCSD2, DcCSD4*, and *DcMSD1*. The expression level of these genes under drought stress had many variations. *DcCSD1, DcCSD2, DcCSD3*, and *DcCSD5* were highly expressed, while *DcCSD4, DcMSD1*, and *DcMSD2* had their expression suppressed (Figure 9).

## Discussion

Plants have a number of effective enzymatic antioxidant defense mechanisms that prevent plant cells from oxidative damage by removing ROS. SOD is one of these antioxidant enzymes that are involved in a variety of plant functions, including growth and providing resistance to environmental stresses by acting as the first line of defense against the harmful effects of high levels of ROS (Feng et al., 2016; Zang et al., 2020; Zhang et al., 2021a). In this study, a genome-wide systematic analysis of the SOD gene family, including sequence phylogeny, conserved motifs, gene structure, chromosomal localization, expression profiling, and structure prediction was carried out in *D. carota*. The SOD gene family is widespread in a variety of plant species such as *Pyrus bretschneideri*, Rosaceae species (Guo et al., 2020), Olive (*Olea europaea L*.) (Zafra et al., 2018), *Larix kaempferi* (Han et al., 2019), barley (*Hordeum vulgare* L., Hv) (Zhang et al., 2021b), *Populus* (Molina-Rueda et al., 2013), *Setaria italica* (Wang et al., 2018), and *S. miltiorrhiza* (Han et al., 2020). In *S. Italica* and *H. vulgare*, SODs were overexpressed under salt stress and drought stress. In melon (*C. melo*) and watermelon (*C. lanatus*), overexpression of SODs under low temperature and salt stress confers resistance to various abiotic stresses (Geng et al., 2018). In cassava (*Manihot esculenta* Crantz), SOD with other enzymes was found to provide resistance against *T. cinnabarinus* as well as abiotic stresses (Lu et al., 2017). This study identified nine SOD genes, which are comparable to the other plants such as *C. sativus* (9) (Zhou et al., 2017), *Solanum lycopersicum* (9) (Feng et al., 2016), *S. bicolor* (8) (Filiz and Tombuloglu, 2015), and *M. truncatula* (7) (Song et al., 2018). These *DcSOD* proteins contained the conserved Cu-ZnSOD domain in CSDs. Furthermore, conserved iron/manganese C-terminal domains were also present in DcFSDs and MSDs, as reported in most of the previous studies such as *H. annuus* L. (Fernández-Ocaña et al., 2011), *C. sinensis* (Zhou et al., 2019), and *C. sativus* (Zhou et al., 2017). These results suggest that this gene family contributes to plant development, structure, and responses to abiotic stresses. Salt, drought, heat, and disease tolerance can be improved by altering the expression of certain genes.

The phylogenetic study revealed that *D. carota* SOD proteins are divided into three distinct subfamilies, namely, Cu/ZnSODs, MnSODs, and FeSODs, which is consistent with *Arabidopsis* (Kliebenstein et al., 1998), *M. truncatula* (Song et al., 2018), *T. aestivum* (Jiang et al., 2019), *H. vulgare* (Zhang et al., 2021b), and *O. sativa* L. (Dehury et al., 2013). On the phylogenetic tree, these three subfamilies were split into two classes, namely, Cu/ZnSODs and Fe-MnSODs. MnSODs and FeSODs were grouped, and a high bootstrap value separated them. This indicates that these genes can be classified according to the type of particular domain they contain and that they may have shared ancestral genes. In cotton, it was identified that MSD and FSD families arose from a common ancestor, whereas the CSD subfamily evolved separately. Thus, the two major groups expanded independently (Wang et al., 2017).

The gene structure was significantly comparable between members of the same subfamily in terms of the number of introns and exons, as well as conserved protein motifs, indicating the phylogenetic relationship of *D. carota* SOD genes was reliable. Moreover, SOD genes from *Arabidopsis, D. carota*, and *O. sativa* have a similar distribution in phylogenetic subgroups and intron–exon organizations, indicating that these genes have been highly conserved throughout evolution. Notably, in *B. marianensis*, a novel SOD has been identified and cloned, which offers significant resistance to high salinity, hydrostatic pressure, chemicals, and adaptability to cold (Li et al., 2020). *OsMSD1/3, AtMSD*, and *DcMSD1/2* had the same motifs. Similarly, *AtCSD1, OsCSD3*, and *DcCS6* had almost the same length and number of exons and introns (Figure 3). These results are comparable to that observed in *C. sativus* and *S. miltiorrhiza*, which contain an average of seven exons. Similar results were found in *T. aestivum* (Jiang et al., 2019), *S. bicolor* (Filiz and Tombuloglu, 2015), and *S. lycopersicum* (Feng et al., 2016). Thus, it is speculated that they may share biological functions. Gain/loss of intron/exon, exonization/pseudoexonization, and insertion/deletion cause exon and intron numbers to vary as well as structural variability in distinct genes (Verma et al., 2019).

By comparing DcSOD proteins to their orthologous proteins in other plants (*A. thaliana* and *G. max*), the evolution of SOD family was examined. In *D. carota, two* pairs of SOD genes originated through segmental duplication. Similarly in *S. bicolor* [54] and watermelon/melon (Kliebenstein et al., 1998), segmental duplication mainly contributed to the expansion of SOD genes. Similarly, SOD genes of *G. raimondii* and *B. napus* also showed segmental duplication, which potentially had a significant part in the expansion of respective plant genomes (Wang et al., 2016; Su et al., 2021). In *S. lycopersicum*, the majority of the genes clustered on a chromosome and experienced tandem duplication. Segmental duplication was also experienced by a few genes. Therefore, in *S. lycopersicum* both types of duplication resulted in the expansion of this gene family (Kuzniak, 2002). In *M. truncatula*, neither tandem nor segmental duplication was found; instead, whole-genome duplication was identified, which caused the generation of these genes (Zang et al., 2020). Similarly, for *Gossypium*, it was proposed that natural selection caused by a number of factors provided the raw material for functional diversification. This resulted in an expansion of this gene family in a relatively short period of time (Lu et al., 2020). This plant experienced the divergence 7–12 Mya, following the duplication of these genes.

In the promoter sequences, *cis*-elements were predicted to gain a better understanding of the role of DcSOD genes under different environmental conditions (Ijaz et al., 2020). Similar to tomato SlSOD genes, different stress, hormone, and light-responsive elements were identified, which were related to drought, anaerobic induction, and low temperature (Zhang et al., 2015). In cotton, elements involved in heat and cold stress responsiveness, MYB binding site, ABA responsiveness, and drought inducibility were found. It showed the obvious involvement of these genes in abiotic stresses and a potentially significant role in antioxidant activity (Lu et al., 2020). In *B. juncea*, several stress-responsive elements such as ARE, W-Box, LTR, WUN, Box-W1, and transcription factor binding sites were found, which were possibly activating the mechanisms involved in stress tolerance. In this way, salt stress was tolerated by the activation of different elements (Verma et al., 2019). Different elements that respond to abscisic acid (ABA) and gibberellin were also found (Supplementary Table S4). Therefore, these findings will help further to understand the diverse functions of DcSOD genes under abiotic stresses.

The 3D structures of these *D. carota* SOD proteins are relatively conserved, which is comparable with conserved domains, phylogeny, and gene structure. These results suggest that *D. carota* SOD genes may play different roles in different tissues of different genotypes. The fundamental structure of CSD is a β-barrel made up of eight antiparallel strands grouped in Greek key patterns, which helps to stabilize the entire protein structure. On the β-barrels' external side, an active metal site is found. MSD and FSDs' secondary and tertiary structures are also inconsistent with the structure of known proteins, and they work together so that SOD takes up either Mn or Fe for its functions (Verma et al., 2019). H_2_O_2_ is a ROS that is harmful to plant growth and development. In contrast, SOD is an antioxidant that is involved in the removal of reactive ROS including H_2_O_2_ (Gill and Tuteja, 2010).

Transcriptome analysis revealed that DcSOD genes had different expression patterns in different tissues and responses to abiotic stresses (dark and light). Similarly, in *B. juncea* and *B. rapa*, the expression of SOD genes under heat and drought stresses was found. The genes of both species, *B. juncea* and *B. rapa*, were overexpressed under drought stress and very few genes were downexpressed. In heat stress, only two genes were upregulated, while most of the genes showed decreased expression (Verma et al., 2019). The SOD genes, especially MSDs in *M. acuminata*, showed specific responses to salt, drought, and heat stresses, which depicted their role in the antioxidant activity (Pal et al., 2013). According to expression analysis, five genes (*DcFSD2, DcFSD3, DcMSD1, DcMSD2*, and *DcCSD2*) have higher expression in xylem and phloem tissues of *D. Carota*. *DcCSD2, DcMSD1*, and *DcMSD2* have higher expression, while *DcFSD2 and DcFSD3* have minor expression. qRT-PCR analysis revealed that some genes from CSD family were highly activated under cold, heat, salt, and drought stresses. Similarly, in grapevine *VvCSD1* was highly expressed in low and high temperatures. Similarly, *VvCSD6* had a higher expression in drought stress. Promoter (*cis*-element) analysis, RNA-seq, and qRT-PCR analysis revealed that most of the genes in the CSD and FSD family, especially *DcCSD1, DcCSD2, DcCSD5*, and *DcFSD2*, had a much higher expression for different abiotic stresses. It is hypothesized that different elements and hormones governed the diverse expression level of DcSODs in different stress as well developmental stages. Therefore, these genes can be used for further research as this study has revealed their important role in abiotic stress.

## Conclusion

In this study, we found 9 SOD genes in *D. carota* and further classified them into three subfamilies, namely, MnSODs (*DcMSD1–2*), FeSODs (*DcFSD2–3*), and Cu/ZnSODs (*DcCSD1–5*), and examined their evolutionary relationships, conserved motifs, gene structure, *cis*-regulatory elements of the promoter regions, and tissue-specific expression pattern. SOD genes have distinct tissue expression levels, indicating that they may have different functions in *D. carota* growth and development. According to *cis*-regulatory elements analysis and expression profiles under different stresses, SOD family genes may be associated with response to hormonal and abiotic stress stimuli. Furthermore, we targeted the SOD genes in response to abiotic stresses (cold, heat, salt, and drought) in different tissues and observed that distinct SOD subfamily genes may play a variety of functions in modulating response to abiotic stresses. qRT-PCR results also revealed their higher expression in various stresses. CSD and FSD families showed higher expression levels for abiotic stresses. Collectively, we laid a basis for further functional characterization of *D. carota* SOD genes in response to abiotic stresses in the future.

## Data Availability Statement

The original contributions presented in the study are included in the article/Supplementary Material, further inquiries can be directed to the corresponding author/s.

## Author Contributions

FA conceived the idea. RZ and KF conducted the experiment and collected data. MS and AR helped in analyzing data. ANS, RZ, and KF helped in initial manuscript preparation. FA, KA, HA, RB, and MA provided technically strengthen the basic idea of research during revision process and helped in funding acquition. SF, FA, and ANS proofread and provided intellectual guidance. All authors read and approved the article.

## Funding

Princess Nourah Bint Abdulrahman University Researchers Supporting Project Number (PNURSP2022R20), Princess Nourah Bint Abdulrahman University, Riyadh, Saudi Arabia.

## Conflict of Interest

The authors declare that the research was conducted in the absence of any commercial or financial relationships that could be construed as a potential conflict of interest.

## Publisher's Note

All claims expressed in this article are solely those of the authors and do not necessarily represent those of their affiliated organizations, or those of the publisher, the editors and the reviewers. Any product that may be evaluated in this article, or claim that may be made by its manufacturer, is not guaranteed or endorsed by the publisher.
