# Supplementary material for: Genome-Wide Characterization of Superoxide Dismutase (SOD) Genes in Daucus carota: Novel Insights Into Structure, Expression, and Binding Interaction With Hydrogen Peroxide (H2O2) Under Abiotic Stress Condition
Source: Front Plant Sci. 2022 Jun 8;13:870241. doi: 10.3389/fpls.2022.870241 (PMC9246500; doi:10.3389/fpls.2022.870241)
Supplement: Supplementary file 1 [file Data_Sheet_1.docx]

**Supplementary files**

**Table S1.** Domains Predictions of DcSOD Proteins.

| **Group** | **Gene Name** | **Gene Symbol** | **Pfam** | **HMMER** | **CDD** | **Interpro** |
| --- | --- | --- | --- | --- | --- | --- |
| FSDs | *DcFSD2* | LOC108220279 | Sod_Fe_C, Sod_Fe_N | Sod_Fe_C, Sod_Fe_N | PLN02685 | Mn/Fe_SOD_N_sf, Mn/Fe_SOD_N, SOD_C_sf, Mn/Fe_SOD, Mn/Fe_SOD_BS, Mn/Fe_SOD_C |
|  | *DcFSD3* | LOC108207726 | Sod_Fe_C, Sod_Fe_N | Sod_Fe_C, Sod_Fe_N | PLN02622 super family | Mn/Fe_SOD_N_sf, Mn/Fe_SOD, Mn/Fe_SOD_C, Mn/Fe_SOD_N, SOD_C_sf, Mn/Fe_SOD_BS |
| MSDs | *DcMSD1* | LOC108223791 | Sod_Fe_C, Sod_Fe_N | Sod_Fe_C, Sod_Fe_N | PLN02471 super family | Mn/Fe_SOD_N_sf, Mn/Fe_SOD_BS, Mn/Fe_SOD_C, Mn/Fe_SOD, SOD_C_sf, Mn/Fe_SOD_N |
|  | *DcMSD2* | LOC108215224 | Sod_Fe_C, Sod_Fe_N | Sod_Fe_C, Sod_Fe_N | PLN02471 super family | Mn/Fe_SOD, Mn/Fe_SOD_BS, SOD_C_sf, Mn/Fe_SOD_N_sf, Mn/Fe_SOD_N, Mn/Fe_SOD_C |
| CDSs | *DcCSD1* | LOC108200536 | Sod_Cu | Sod_Cu | Cu-Zn_Superoxide_Dismutase super family | SOD_Cu_Zn_dom, SOD-like_Cu/Zn_dom_sf, SOD_Cu/Zn_BS, SOD_Cu/Zn_/chaperone |
|  | *DcCSD2* | LOC108219531 | Sod_Cu | Sod_Cu | Cu-Zn_Superoxide_Dismutase super family | SOD_Cu_Zn_dom, SOD_Cu/Zn_/chaperone, SOD_Cu/Zn_BS, SOD-like_Cu/Zn_dom_sf |
|  | *DcCSD3* | LOC108222825 | Sod_Cu | Sod_Cu | Cu-Zn_Superoxide_Dismutase super family | SOD-like_Cu/Zn_dom_sf, SOD_Cu_Zn_dom, SOD_Cu/Zn_/chaperone , SOD_Cu/Zn_BS |
|  | *DcCSD4* | LOC108226548 | Sod_Cu | Sod_Cu | Cu-Zn_Superoxide_Dismutase super family | SOD_Cu/Zn_BS, SOD-like_Cu/Zn_dom_sf , SOD_Cu_Zn_dom, SOD_Cu/Zn_/chaperone |
|  | *DcCSD5* | LOC108212562 | Sod_Cu | Sod_Cu | Cu-Zn_Superoxide_Dismutase super family | SOD_Cu_Zn_dom, SOD-like_Cu/Zn_dom_sf , SOD_Cu/Zn_BS, SOD_Cu/Zn_/chaperone |

**Table S2.** Ka/Ks values and duplication time calculation in MYA.

| **Duplicated Gene Pairs** | **ka** | **ks** | **ka/ks** | **Time (MYA*)** | **Duplication type** |
| --- | --- | --- | --- | --- | --- |
| AtCSD2/GmCSD3 | 0.1937 | 0.4994 | 0.387865 | 16.64666667 | Segmental |
| AtCSD2/GmCSD2 | 0.1932 | 0.5584 | 0.345989 | 18.61333333 | Segmental |
| AtCSD2/SlSOD3 | 0.197 | 0.5512 | 0.357402 | 18.37333333 | Segmental |
| AtFSD1/GMFSD2 | 0.329 | 0.6381 | 0.515593 | 21.27 | Segmental |
| AtMSD/GmMSD2 | 0.2672 | 0.4717 | 0.566462 | 15.72333333 | Segmental |
| DcFSD3/SlSOD7 | 0.1999 | 0.4498 | 0.44442 | 14.99333333 | Segmental |
| DcCSD1/DcCSD4 | 0.0804 | 0.1077 | 0.746518 | 3.59 | Segmental |
| DcCSD2/GmCSD3 | 0.1827 | 0.4395 | 0.4157 | 14.65 | Segmental |
| DcCSD2/GmCSD2 | 0.1728 | 0.4947 | 0.349303 | 16.49 | Segmental |
| DcCSD2/SlSOD3 | 0.1574 | 0.389 | 0.404627 | 12.96666667 | Segmental |
| DcCSD3/GmCSD5 | 0.2394 | 0.376 | 0.636702 | 12.53333333 | Segmental |
| DcMSD1/DcMSD2 | 0.0774 | 0.2344 | 0.330205 | 7.813333333 | Segmental |
| GmMSD1/GmMSD2 | 0.0113 | 0.0274 | 0.412409 | 0.913333333 | Segmental |
| GmFSD3/GmFSD5 | 0.0307 | 0.0692 | 0.443642 | 2.306666667 | Segmental |
| GmFSD3/GmFSD1 | 0.1283 | 0.2258 | 0.568202 | 7.526666667 | Segmental |
| GmFSD3/GmFSD2 | 0.1325 | 0.1914 | 0.692268 | 6.38 | Segmental |
| GmFSD5/GmFSD1 | 0.1467 | 0.205 | 0.71561 | 6.833333333 | Segmental |
| GmFSd5/GmFSD2 | 0.1601 | 0.1549 | 1.03357 | 5.163333333 | Segmental |
| GmFSD1/GMFSD2 | 0.0497 | 0.0431 | 1.153132 | 1.436666667 | Segmental |
| GmCSD1/GmCSD6 | 0.0514 | 0.0926 | 0.555076 | 3.086666667 | Segmental |
| GmCSD4/GmCSD3 | 0.1576 | 0.2908 | 0.541953 | 9.693333333 | Segmental |
| GmCSD4/GmCSD2 | 0.1504 | 0.3237 | 0.464628 | 10.79 | Segmental |
| GmCSD3/GmCSD2 | 0.0075 | 0.0693 | 0.108225 | 2.31 | Segmental |
| GmCSD3/SlSOD3 | 0.1475 | 0.5638 | 0.261618 | 18.79333333 | Segmental |
| GmCSD2/SlSOD3 | 0.1382 | 0.5996 | 0.230487 | 19.98666667 | Segmental |
| SlSOD5/SlSOD6 | 0.1628 | 0.3873 | 0.420346 | 12.91 | Segmental |
| SlSOD5/SlSOD8 | 0.0758 | 0.2384 | 0.317953 | 7.946666667 | Tendem |
| SISOD6/SISOD8 | 0.1948 | 0.3484 | 0.559127 | 11.61333333 | Segmental |

**Table S3.** Reactive oxygen species i.e., hydrogen peroxide (H_2_O_2)_ docked against nine *DcSODs*, screened by molecular docking studies and evaluated by PatchDock server.

| **Protein Name** | **Global Energy**  **(****Kcal/Mol)** | **Attractive VdW**  **(****Kcal/Mol)** | **Bond Distance**  **(Å)** | **Conserved Residue** |
| --- | --- | --- | --- | --- |
| DcFSD2 | -3.51 | -2.04 | 2.998 | ARG 35 |
|  |  |  | 2.594 | GLN 38 |
|  |  |  | 2.584 | GLU 46 |
|  |  |  | 3.067 | LEU 47 |
| DcFSD3 | -1.94 | -2.19 | 2.460 | ASP 50 |
|  |  |  | 2.324 | GLY 56 |
| DcMSD1 | -3.57 | -2.78 | 2.843 | ALA 33 |
|  |  |  | 2.269 | TRP 103 |
|  |  |  | 2.329 | LYS 104 |
| DcMSD2 | -3.13 | -2.05 | 2.518 | TRP 210 |
|  |  |  | 2.159 | TRP 215 |
|  |  |  | 2.772 | VAL 136 |
| DcCSD1 | -3.29 | -1.85 | 2.792 | ASN 85 |
|  |  |  | 2.240 | VAL 86 |
|  |  |  | 2.848 | PHE 96 |
| DcCSD2 | -3.38 | -3.28 | 3.085 | THR 160 |
|  |  |  | 2.870 | GLN 161 |
|  |  |  | 2.377 | PRO 163 |
| DcCSD3 | -2.14 | -1.41 | 2.098 | GLY 12 |
|  |  |  | 1.086 | ASP 13 |
| DcCSD4 | -3.69 | -1.93 | 2.356 | SER 11 |
|  |  |  | 3.536 | CYS 56 |
|  |  |  | 2.793 | ARG 142 |
|  |  |  | 3.138 | VAL 143 |
| DcCSD5 | -3.06 | -2.02 | 3.108 | GLY 36 |
|  |  |  | 2.329 | LEU 37 |
|  |  |  | 2.363 | ILE 143 |

**Table S4.** Promoter analysis of *SOD* genes.

| **Gene name** | **Cis-element** | **Number of Cis Element** | **Function** |
| --- | --- | --- | --- |
| *DcFSD2* | LAMP-element | 2 | part of a light responsive element |
|  | ABRE | 1 | cis-acting element involved in the abscisic acid responsiveness |
|  | TCT-motif | 2 | part of a light responsive element |
|  | chs-CMA2a | 1 | part of a light responsive element |
|  | G-Box | 1 | cis-acting regulatory element involved in light responsiveness |
|  | LTR | 1 | cis-acting element involved in low-temperature responsiveness |
| *DcFSD3* | GATA-motif | 1 | part of a light responsive element |
|  | TCA-element | 1 | cis-acting element involved in salicylic acid responsiveness |
|  | Box 4 | 1 | part of a conserved DNA module involved in light responsiveness |
|  | GT1-motif | 1 | light responsive element |
|  | ARE | 1 | cis-acting regulatory element essential for the anaerobic induction |
| *DcMSD1* | O2-site | 2 | cis-acting regulatory element involved in zein metabolism regulation |
|  | GATA-motif | 2 | part of a light responsive element |
|  | G-Box | 2 | cis-acting regulatory element involved in light responsiveness |
|  | CGTCA-motif | 1 | cis-acting regulatory element involved in the MeJA-responsiveness |
|  | Box 4 | 1 | part of a conserved DNA module involved in light responsiveness |
|  | TGACG-motif | 1 | cis-acting regulatory element involved in the MeJA-responsiveness |
|  | ABRE | 2 | cis-acting element involved in the abscisic acid responsiveness |
|  | GC-motif | 1 | enhancer-like element involved in anoxic specific inducibility |
| *DcMSD2* | GT1-motif | 3 | light responsive element |
|  | P-box | 1 | gibberellin-responsive element |
|  | G-box | 1 | cis-acting regulatory element involved in light responsiveness |
|  | ACE | 1 | cis-acting element involved in light responsiveness |
|  | ABRE | 2 | cis-acting element involved in the abscisic acid responsiveness |
|  | GCN4_motif | 1 | cis-regulatory element involved in endosperm expression |
|  | Box 4 | 1 | part of a conserved DNA module involved in light responsiveness |
|  | AAAC-motif | 2 | light responsive element |
|  | circadian | 1 | cis-acting regulatory element involved in circadian control |
|  | AuxRR-core | 1 | cis-acting regulatory element involved in auxin responsiveness |
|  | MRE | 1 | MYB binding site involved in light responsiveness |
|  | G-Box | 1 | cis-acting regulatory element involved in light responsiveness |
| *DcCSD1* | TCA-element | 1 | cis-acting element involved in salicylic acid responsiveness |
|  | GATA-motif | 1 | part of a light responsive element |
|  | Box 4 | 4 | part of a conserved DNA module involved in light responsiveness |
|  | MBS | 1 | MYB binding site involved in drought-inducibility |
|  | LTR | 3 | cis-acting element involved in low-temperature responsiveness |
|  | TGA-element | 1 | auxin-responsive element |
|  | ARE | 6 | cis-acting regulatory element essential for the anaerobic induction |
| *DcCSD2* | Box 4 | 1 | part of a conserved DNA module involved in light responsiveness |
|  | ACE | 1 | cis-acting element involved in light responsiveness |
|  | GA-motif | 1 | part of a light responsive element |
|  | TATC-box | 1 | cis-acting element involved in gibberellin-responsiveness |
|  | AE-box | 1 | part of a module for light response |
| *DcCSD3* | GATA-motif | 1 | part of a light responsive element |
|  | I-box | 1 | part of a light responsive element |
|  | TCT-motif | 2 | part of a light responsive element |
| *DcCSD4* | G-Box | 1 | cis-acting regulatory element involved in light responsiveness |
|  | ARE | 1 | cis-acting regulatory element essential for the anaerobic induction |
|  | MBS | 1 | MYB binding site involved in drought-inducibility |
|  | chs-CMA2a | 8 | part of a light responsive element |
|  | G-box | 2 | cis-acting regulatory element involved in light responsiveness |
|  | AE-box | 1 | part of a module for light response |
|  | Box 4 | 1 | part of a conserved DNA module involved in light responsiveness |
|  | ABRE | 1 | cis-acting element involved in the abscisic acid responsiveness |
| *DcCSD5* | TCA-element | 1 | cis-acting element involved in salicylic acid responsiveness |
|  | ABRE | 2 | cis-acting element involved in the abscisic acid responsiveness |
|  | MBS | 1 | MYB binding site involved in drought-inducibility |
|  | CGTCA-motif | 2 | cis-acting regulatory element involved in the MeJA-responsiveness |
|  | ARE | 1 | cis-acting regulatory element essential for the anaerobic induction |
|  | G-Box | 6 | cis-acting regulatory element involved in light responsiveness |
|  | TGACG-motif | 2 | cis-acting regulatory element involved in the MeJA-responsiveness |
|  | CAT-box | 2 | cis-acting regulatory element related to meristem expression |
|  | G-box | 1 | cis-acting regulatory element involved in light responsiveness |
|  | I-box | 2 | part of a light responsive element |
|  | ACE | 1 | cis-acting element involved in light responsiveness |

**Table S5.** List of primers used in this study.

| **Gene Name** | **Primer Sequence** |  | **Product Length** |
| --- | --- | --- | --- |
| *DcFSD2* | AGTTGGCAGCATCATCTCAGT | F | 83 |
|  | TTTGGGGGAGGAATGGAAGC | R |  |
| *DcFSD3* | GGCGGTCATCAGCCTGTATT | F | 108 |
|  | ACCAAATAGTGTCAGTGCTGC | R |  |
| *DcMSD1* | CGGCGATTAGCGGTGAGATA | F | 117 |
|  | TAGAGTCAGACCGGGAGACA | R |  |
| *DcMSD2* | TTACCAAGAAGAGCGTGCGG | F | 118 |
|  | TCACCACTAATTGCCGGCTC | R |  |
| *DcCSD1* | TCACCAGCATGACGGTTCTC | F | 80 |
|  | TGTTAATCTGTAGGACCCCACT | R |  |
| *DcCSD2* | ACACACCTGCATTCAAAAGGG | F | 119 |
|  | CCTGCCATCGTTGTCTTGTTG | R |  |
| *DcCSD3* | AGGCCAGATCTTGCGTTTGT | F | 78 |
|  | TGCTGCCCCATAGTGATGTT | R |  |
| *DcCSD4* | ATTTAAATCTGCAGGACCCCAT | F | 71 |
|  | GGCGGTTCTCATCTTCTGGG | R |  |
| *DcCSD5* | TGCGCCTGATGATGAAATTCG | F | 70 |
|  | TCACATACCGTCATGGCCAACT | R |  |
