# Supplementary figures and images for: Genome-Wide Characterization of Superoxide Dismutase (SOD) Genes in Daucus carota: Novel Insights Into Structure, Expression, and Binding Interaction With Hydrogen Peroxide (H2O2) Under Abiotic Stress Condition
Source: Front Plant Sci. 2022 Jun 8;13:870241. doi: 10.3389/fpls.2022.870241 (PMC9246500; doi:10.3389/fpls.2022.870241)

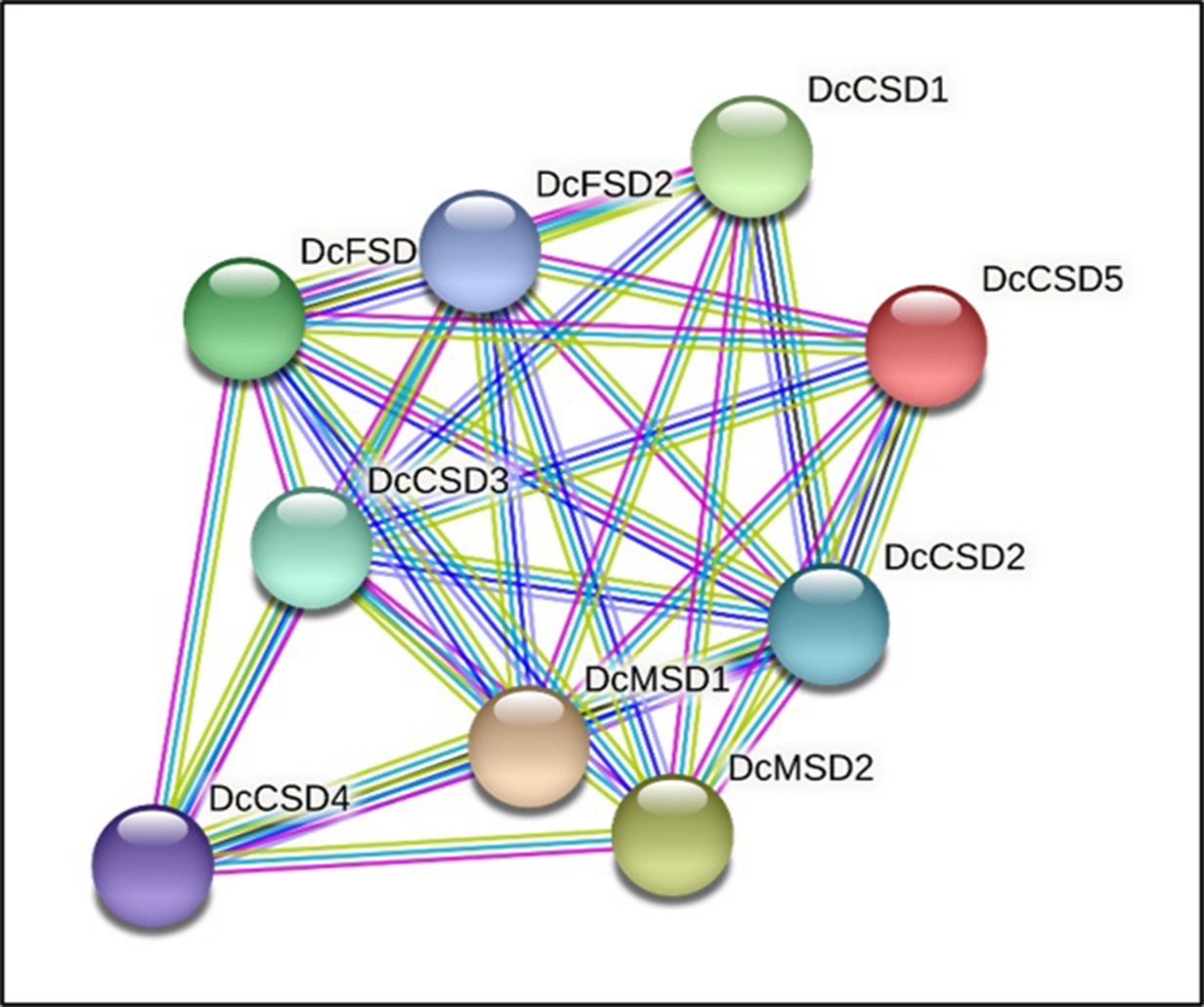

Supplement: Supplementary file 2 [file Image_1.JPEG]
